# Responsive Hyaluronic Acid–Ethylacrylamide Microgels Fabricated Using Microfluidics Technique

**DOI:** 10.3390/gels8090588

**Published:** 2022-09-15

**Authors:** Marcus Wanselius, Agnes Rodler, Sean S. Searle, Susanna Abrahmsén-Alami, Per Hansson

**Affiliations:** 1 Department of Medicinal Chemistry, Uppsala University, SE-751 23 Uppsala, Sweden; 2 Innovation Strategies & External Liaison, Pharmaceutical Technology & Development, Operations, AstraZeneca, SE-431 83 Gothenburg, Sweden

**Keywords:** microsphere, microgel, synthesis, microfluidics, hyaluronic acid, responsiveness, swelling, permeability, partition coefficient, dextran, confocal microscopy

## Abstract

Volume changes of responsive microgels can probe interactions between polyelectrolytes and species of opposite charges such as peptides and proteins. We have investigated a microfluidics method to synthesize highly responsive, covalently crosslinked, hyaluronic acid microgels for such purposes. Sodium hyaluronate (HA), pre-modified with ethylacrylamide functionalities, was crosslinked in aqueous droplets created with a microfluidic technique. We varied the microgel properties by changing the degree of modification and concentration of HA in the reaction mixture. The degree of modification was determined by ^1^H NMR. Light microscopy was used to investigate the responsiveness of the microgels to osmotic stress in aqueous saline solutions by simultaneously monitoring individual microgel species in hydrodynamic traps. The permeability of the microgels to FITC-dextrans of molecular weights between 4 and 250 kDa was investigated using confocal laser scanning microscopy. The results show that the microgels were spherical with diameters between 100 and 500 µm and the responsivity tunable by changing the degree of modification and the HA concentration. Microgels were fully permeable to all investigated FITC-dextran probes. The partitioning to the microgel from an aqueous solution decreased with the increasing molecular weight of the probe, which is in qualitative agreement with theories of homogeneous gel networks.

## 1. Introduction

Hyaluronic acid (HA) is a biopolymer that is well suited as an excipient in drug formulations intended for injection into the body, as filler material in cosmetic and medical surgery, and as scaffoldings for cell growth in tissue engineering [1,2,3]. The reason is that it combines excellent biocompatibility with useful polyelectrolyte properties [4] such as ion binding and a capacity to absorb and retain water. HA is a major component of the extracellular matrix in living tissues, where the water-retaining property is of vital importance [5]. The principle is that the carboxylic acid groups on the polymer are deprotonated at physiological pH, and to maintain electroneutrality, the chains become associated with counterions, providing an osmotic swelling pressure to the tissue. The ion-binding property is particularly useful in drug delivery, where linear and crosslinked forms of the negatively charged polymer can serve as carriers of cationic self-assembling drugs or cationic protein and peptide drugs [6,7,8,9].

Proteins and molecular self-assemblies carrying high positive charges readily form polyelectrolyte complexes with anionic polyions. Depending on the interaction strength and method of preparation, such complexes form colloidal suspensions (“particles”), liquid coacervates, or water-poor complex phases (“precipitates”) [10,11,12,13,14,15]. Typically, the complexes are stable at a low ionic strength but dissolve at an elevated ionic strength or at pH values where the polyion and/or protein have a low charge. In applications in drug delivery, this reversibility is useful since the physiological ionic strength is often high enough to trigger protein release from the formulation. Responsive polyelectrolyte microgels are interesting in this respect because they swell upon the release of the protein drug load. The volume change can be utilized as a means to control the release rate [16]. Transarterial chemoembolization (TACE) is a method for treating liver cancer, where microbeads loaded with doxorubicin or related substances are administered directly to the blood vessels feeding the tumor [17,18,19,20,21,22]. In this case, the swelling of the beads clogs the blood vessels, which is beneficial because it limits the systemic spreading of the highly toxic drug and improves treatment by stopping the blood flow to the tumor.

The swelling response of microgels can also be used as a method for studying the interaction between polymers and various types of species including multivalent ions, surfactants, peptides, and proteins [16,23,24,25,26,27,28,29]. In our laboratory, we have developed micropipette-assisted microscopy techniques for this purpose based on the responsiveness of large spherical microgel networks (“microspheres”) [24,26,27]. Recently, we have developed a microfluidics platform for the investigation of microspheres confined to hydrodynamic traps [30], which allows simultaneous monitoring of a large number of microspheres. Both methods can be used to measure the strength of the interaction between the network polyelectrolytes and charged species simply by monitoring the volume response of the microspheres. We recently proposed this as a method for investigating how strongly protein and peptide drugs interact with biopolymers [30].

The latter aspect is important in subcutaneous drug delivery, where macromolecular drugs injected into the adipose tissue need to pass through the extracellular matrix in order to be absorbed by the circulatory system via blood capillaries or lymphatic vessels [31,32,33,34,35]. Experiments have shown that positively charged therapeutic proteins are absorbed at a lower rate than net negatively charged proteins. This suggests that the electrostatic interaction with the negatively charged polymers present in the ECM reduces the transport rate of positively charged proteins. However, the drug absorption rates and fractions absorbed after subcutaneous administration in humans have turned out to be notoriously difficult to predict from animal in vivo data [32,34]. This has created an acute need for in vitro methods for testing new protein and peptide-based drug products during the development phase [36,37,38,39]. Very recently, we showed that our microfluidics-based method was able to rank three model substances with respect to the strength of the interaction with HA and two other polyelectrolytes [30]. In future work, we will investigate whether the in vitro data on the interaction strength between HA and protein/peptide drugs provided by this method can be correlated with in vivo absorption rates and bioavailability.

The performance of the method depends on the characteristics of the microspheres. The networks must be highly responsive and have a large diameter (50–500 µm) to allow for accurate microscopy studies. Furthermore, they should have a large network mesh size, homogeneous composition, and chemical and mechanical stability, and the network chains should maintain the essential physicochemical properties of native HA. The aim of the present work is to fabricate HA microgels fulfilling these criteria. Attempts in our lab to use conventional inverse emulsion methodology with the crosslinker entering from the homogeneous phase resulted in heterogeneous microgels. Others have fabricated HA microgels using spray-drying and solvent evaporation techniques [40,41,42,43,44]. The resulting microgels were either heterogeneous or too small to suit our purposes (see above). Recently, Heida et al. [45] showed that HA microgels with well-controlled physicochemical and mechanical properties can be produced with a microfluidics-based method. They used click chemistry reactions to crosslink modified HA derivatives using homobifunctional poly(ethylene glycol) (PEG) crosslinkers. The approach made it possible to systematically vary the properties of the microgels. In our work, we have used a similar inverse emulsion droplet microfluidic-assisted setup to create HA-filled aqueous droplets, followed by crosslinking of the HA chains in a subsequent step. However, for the microgel application we are aiming for (i.e., probing interactions with HA), it is crucial that the crosslinker alters the solution properties of HA as little as possible. We have therefore selected ethylacrylamide as a crosslinker, which has a much lower molecular weight than the ones used by Heida et al.

In this paper, we first show that HA can be prepared with different degrees of ethylacrylamide modification and subsequently crosslinked in aqueous droplets by a UV-initiated reaction. Then, by exposing the microgels to solutions of different ionic strengths, we show that the responsivity of the network can be tuned by varying the degree of modification of HA and the concentration of HA in the reaction mixture during crosslinking. Finally, we demonstrate that the microgel networks are penetrable to dextran diffusion probes with molecular weights comparable to those of antibodies. The results show that they are well suited for the intended application to demonstrate the interaction between HA and therapeutic proteins.

## 2. Results and Discussion

**Ethylacrylamide modification of HA and microgel synthesis.** Synthesis of ethylacrylamide-modified HA resulted in four batches with different degrees of modification. Equivalent ratios of precursors, the targeted degree of modification ($f_{0}$), and the actual resulting degree of modification (*f*) experimentally determined by ^1^H NMR are presented in Table 1. The results show that both the ratio of N-(2-aminoethyl) acrylamide hydrochloride to HA and the reaction time affected the degree of modification. Table 2 shows the HA degree of modification and the amount of HA in the aqueous solution during microgel production for each microgel batch. For simplicity, we denote each batch by a number for the rest of this work. The microgels were fabricated using a custom-built microfluidic chip for droplet production (MDP) as described in the Section 4.

**Figure 1:**
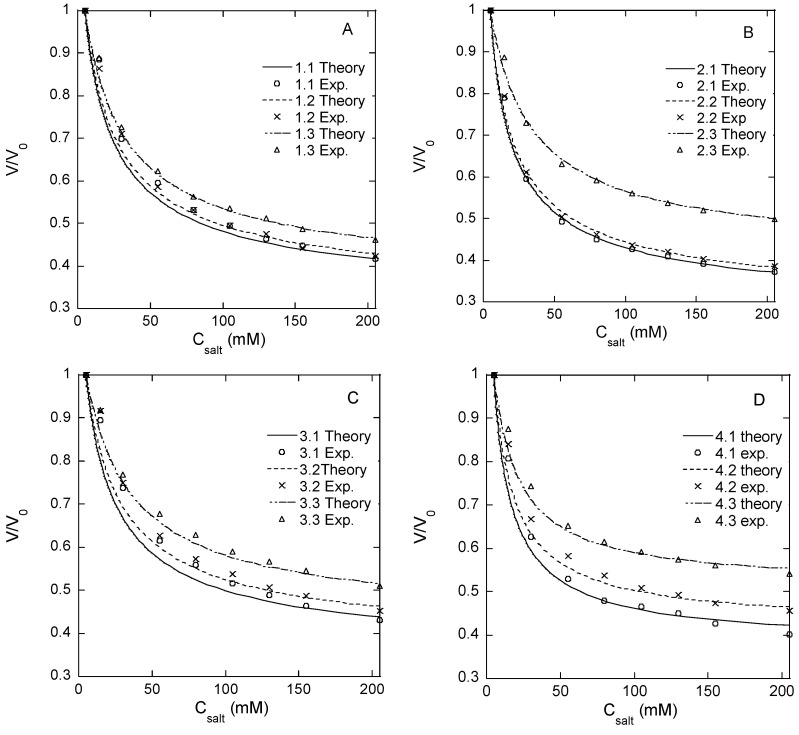
Volume change of HA microgels at exposure to different concentrations of NaCl in PB, flow rate 200 µL/min. Degrees of modification: 13% (**A**), 21% (**B**), 21% (**C**), 33% (**D**). Experimental data (symbols) and model fits (curves) for each microgel batch are indicated in the figure legends.

**Responsivity of microgel.** Microgels confined to hydrodynamic traps on a microfluidic chip (see below) were exposed to phosphate buffer solutions of different NaCl concentrations in a range from zero to 1 M. We use PB to denote the phosphate-buffered media containing sodium phosphate monobasic (3.5 mM) and sodium phosphate dibasic (1.5 mM), which are used as the standard aqueous medium in all microfluidic experiments. The volume of the microgels at each NaCl concentration was determined from the measurements of the microgel diameter. The results are summarized in Figure 1A–D and Table 2, where *V* is the actual microgel volume and *V_0_* is the volume in the PB with no NaCl added. Each volume ratio presented is the mean value of eight different microgels in the same microfluidic chip. Table 2 shows the ratio of the volume determined at 1 M NaCl, where the ionic swelling pressure is largely removed, and the volume at 0 M NaCl where the ionic swelling pressure is substantial (swelling ratios at intermediate NaCl concentrations are given in Table S1). The smaller the volume ratio (*V*/*V_0_*), the larger the volume response. The standard deviation of *V*/*V_0_* is small (Table 1), showing that the relative volume change varied little among the microgels from the same batch. Since *V_0_* varied among the individual species, the results also show that the responsivity was practically independent of the absolute size of the microgel.

**Figure 2:**
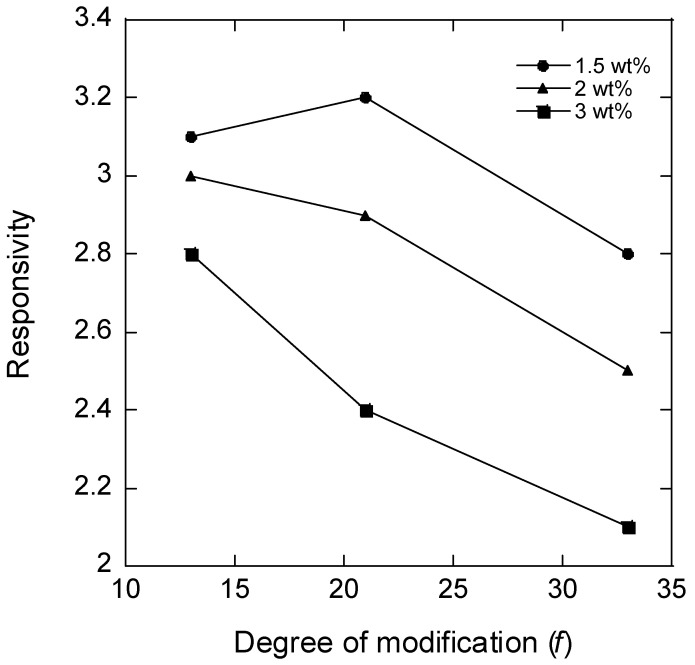
Responsivity plotted vs. degree of modification for microgels crosslinked at different concentrations of HA (as indicated).

As a measure of “responsivity”, we use the inverse volume ratio *V_0_*/*V* (Table 2). Figure 2 shows a plot of the responsivity vs. the degree of modification of HA (*f*) for microgels with different HA concentrations in the reaction mixture during microgel synthesis; the plotted values for *f* = 21% are the means of the responsivity for batches 2 and 3. According to the theory of polyelectrolyte gels, the responsivity should decrease with the increasing degree of crosslinking between the chains and the increasing polymer concentration in the solution during crosslinking (larger elastic modulus), and the decreasing fraction of the charged segments on the network chains (weaker ionic swelling pressure). The data in Figure 2 are broadly consistent with the expected behavior. Thus, for microgels with the same degree of modification, the responsivity decreased with the increasing HA concentration in the reaction mixture. This was expected since the concentration of elastically active chains in the network should increase both by the concentration increase as such and also by the increased probability of two modified segments coming into contact to form a crosslink. Furthermore, the responsivity decreased with the increasing degree of modification as expected since increasing the concentration of modifications should increase the crosslinking density and decrease the charge of the network. However, at the lowest degree of modification, the responsivity depended less on the HA concentration in the reaction mixture than at higher degrees of modification, and for the lowest HA concentration, the responsivity varied non-monotonically with the degree of modification.

To understand this better, we attempted to estimate how much the change in the degree of modification actually led to a change in the (apparent) degree of crosslinking. This was done by fitting a modified version of the Flory–Rehner theory [46] to the experimental data in Figure 1A–D. According to the theory, the osmotic pressure difference between the microgel and the electrolyte solution is considered to have three independent contributions:

(1) $$\Delta \Pi =\Pi_{FH}+{\Delta \Pi }_{ion}+\Pi_{def}$$

The first derives from the free energy of mixing the network chains with the solvent as described by the Flory–Huggins polymer solution theory [47]:

(2) $$\Pi_{FH}=-\frac{RT}{{\bar{v}}_{w}}\left(\ln\left(1-\varphi \right)+\varphi +\chi \varphi^{2}\right)$$

*R* is the ideal gas constant, *T* is the absolute temperature, ${\bar{v}}_{w}$ is the molar volume of water, φ is the polymer volume fraction in the microgel, and χ is the Flory–Huggins polymer–solvent interaction parameter.

The second term in Equation (1) is the contribution from the free energy of mixing the mobile ions. By treating the aqueous solution as ideal and neglecting counterion binding to the network chains, the osmotic pressure difference can be written [48]:

(3) $${\Delta \Pi }_{ion}=2RTC_{salt}(\sqrt{{\left(\frac{\left(1-f\right)C_{p}}{2C_{salt}}\right)}^{2}+1}-1)$$

$C_{salt}$ is the concentration of the 1:1 electrolyte in the bulk aqueous solution, and $C_{p}$ is the concentration of the HA disaccharide (“monomer”) units in the microgel. Equation (3) accounts for the non-uniform distribution of salt between the microgel and the solution (Donnan equilibrium).

The third term in Equation (1) is the contribution from the elastic deformation-free energy of the network. We use the inverse Langevin theory and write the contribution as a series expansion [49,50]:

(4) $$\Pi_{def}=\frac{RT\varphi }{M{\bar{v}}_{ss}}\left\{\frac{1}{2}-{\left(\frac{\varphi_{0}}{\varphi }\right)}^{\frac{2}{3}}-\frac{3}{5M}{\left(\frac{\varphi_{0}}{\varphi }\right)}^{\frac{4}{3}}-\frac{99}{175M^{2}}{\left(\frac{\varphi_{0}}{\varphi }\right)}^{2}-\frac{513}{875M^{3}}{\left(\frac{\varphi_{0}}{\varphi }\right)}^{\frac{8}{3}}+\ldots \right\}$$

*M* is the number of statistical segments between crosslinks, ${\bar{v}}_{ss}$ is the molar volume of a statistical segment in the chain, and $\varphi_{0}$ is the volume fraction of polymer in a reference state of the microgel, taken to be equal to the polymer volume fraction in the solution where the crosslinking reaction took place.

Equations (1)–(4) provide a mathematical relationship between the relative volume of the microgel and the salt concentration in the solution valid at equilibrium (ΔΠ=0). We fitted the relationship to the experimental data with *M* as a fitting parameter. We used the value 7.11 × 10^−3^ mole/m^3^ for the molar volume per statistical segment (${\bar{v}}_{ss}$), corresponding to the volume of 15 disaccharide units of a length of 1.0 nm [4,51]; the molar volume of water was set to 1.80 × 10^−5^ mole/m^3^; the concentration of salt was equal to that used in each experiment; the polymer volume fractions in the reference state of the network ($\varphi_{0}$) were 0.018, 0.024, and 0.035 for the microgels prepared with 1.5, 2, and 3% *w*/*w* HA, respectively; the molar concentrations of HA disaccharide units and polymer volume fractions were calculated using 1.0 g/mL as the density of the microgels; the degree of modification (*f*) was set equal to the experimentally determined values given in Table 2; the Flory–Huggins interaction parameter for unmodified HA ($\chi_{0}=0.439$) was taken from the literature [52]. The model fits are represented by the curves in Figure 1A–D; the resulting *M* values and the corresponding number of disaccharide units between crosslinks are given in Table 2. The results suggest that the chain length between crosslinks and thus the actual degree of crosslinking depended little on the degree of modification, meaning that the yield of the crosslinking reaction decreased with the increasing degree of modification of HA. For the microgels with the lowest degree of modification, the results imply that only about one functional group out of four was involved in a crosslink, and for the microgels with higher degrees of modification, the fraction was even smaller. This is in agreement with the observed high responsivity. In fact, a larger fraction of modifications taking part in crosslinks would have been incompatible with the observed responsivity of the microgels with the highest degree of modification. The HA concentrations (1.5–3 wt%) at which the crosslinking reaction was carried out were clearly above the overlap concentration of HA for the molecular weight used, a condition that must be fulfilled for intact networks to form. However, since HA chains are only semi-flexible [51], geometric considerations suggest that only a fraction of the modifications should be able to form crosslinks between the more highly modified HA chains. A similar effect was reported by Heida et al. for HASA microgels consisting of HA chains functionalized by 3-(2-pyridyldithio) propionyl hydrazide connected by PEG-based crosslinks [45].

When evaluating the above results, one should keep in mind that the elasticity model (Equation (4)) first derived and tested for rubber materials [50] is only expected to provide *M* values of the correct order of magnitude, even for networks where effects of entanglements and “dangling chains” are minor. Furthermore, the model applies strictly only when *M* is substantially larger than unity. However, it is fully realistic to imagine a network with an average of 10–20 disaccharide units between crosslinks, as the results of the model fits suggest (cf. Table 2).

In conclusion, the salt response study highlights the high responsivity of the present microgels and shows that the preparation method allows the responsivity to be tuned by the variation of the HA modification degree and concentration in the solution during crosslinking. However, the origin of the non-monotonic variation of the responsivity with the degree of modification for the microgels prepared at 1.5 wt% HA remains unclear. If the actual degree of crosslinking changed only marginally, as suggested by the results of the model fit, the non-monotonic variation must be attributed to other effects of modifying the chains such as the charge density or change in the solvency of the network caused by the chemical nature of the attached group.

**Figure 3:**
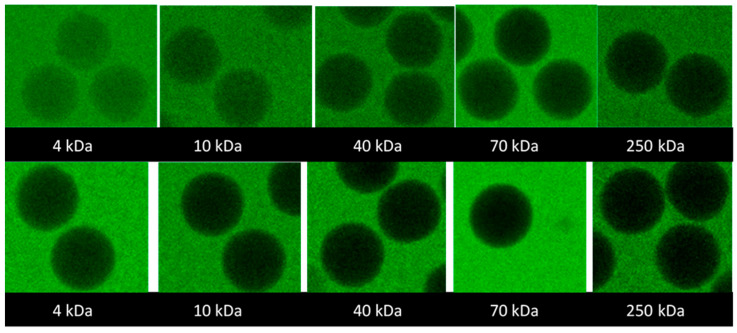
CLSM false-colour images of central focal plane of microgels 2.1 (upper) and 2.3 (lower) in equilibrium with aqueous solutions of different-sized FITC-dextran. Size of microgels ~130–150 µm in diameter.

**Permeability to dextran probes.** The possibility of proteins of different sizes diffusing into the microgels and the homogeneity of the network are other important characteristics of microgels intended for studies of the interaction between polymers and proteins. We employed a series of FITC-labeled dextrans to investigate the permeability of the microgels to solutes of different sizes. The molecular weights (M_w_) of the dextran probes used are given in Table 3 together with the hydrodynamic radii. According to the manufacturer, they were slightly branched, and fractions with M_w_ 2–10 kDa should behave as expandable coils in an aqueous solution and fractions with M_w_ > 10 kD as highly branched. Figure 3 shows the representative confocal laser scanning microscopy (CLSM) images of the microgels taken after seven days of equilibration in aqueous solutions containing 2 mg/mL probes. The full set of images is provided in the Supplementary Materials S2; microgels 3.1, 3.2, and 3.3 were excluded from the study because their modification and salt response were similar to microgels 2.1, 2.2, and 2.3. The focal plane was through the center of the microgels. The fluorescence intensity was lower than in the surrounding solution and decreased with the increasing molecular weight of the probe. However, the intensity was uniformly distributed over the focal plane even for the probe with the highest molecular weight. The results suggest that all investigated probes were uniformly distributed inside the microgels (at least on a macroscopic scale) but the partitioning to the microgels decreased with the increasing molecular weight.

**Figure 4:**
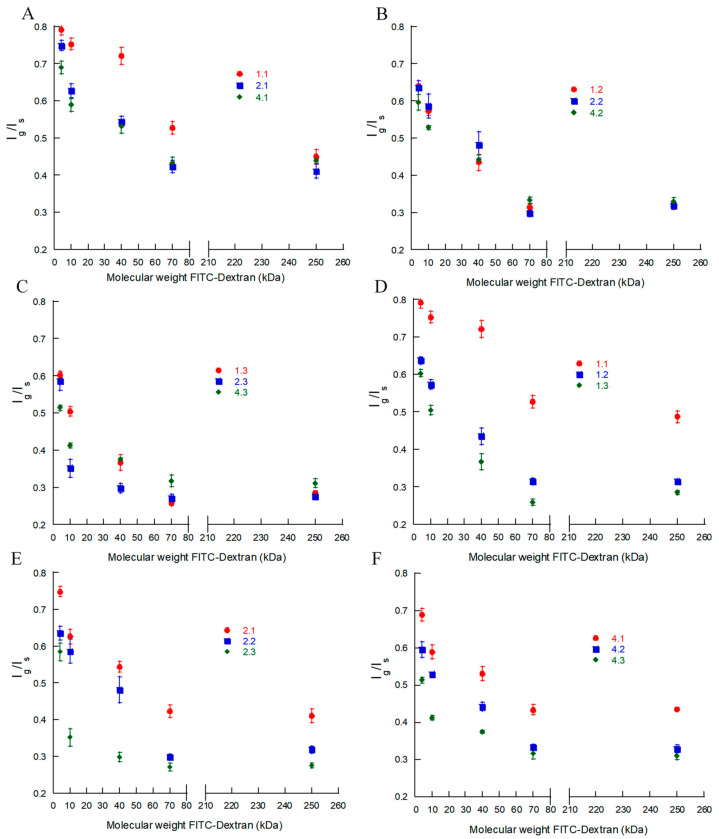
Fluorescence intensity ratio of the central focal plane of the microgels and surrounding 2 mg/mL FITC-dextran solution. Comparison of functionalization degree of HA in microgel at (**A**): 1.5% *w*/*w* HA, (**B**): 2% *w*/*w* HA, and (**C**): 3% *w*/*w* HA in solution during gel production. Comparison of different amounts (% *w*/*w*) of HA in solution during microgel production at (**D**): 13% (**E**): 21%, and (**F**): 33% ethylacrylamide modification of HA. All values presented are the mean of the nine individual microgels.

To quantify the observed effects, we compared the fluorescence intensity at an excitation/emission wavelength of 488/520 nm in the center plane of the microgels (*I_g_*) and that in the surrounding solution (*I_s_*). Figure 4A–F show the intensity ratio *I_g_/I_s_* as a function of the molecular weight of the probe for each batch in Table 2. Each data point is the mean value for the nine different microgels provided in Table S2. In what follows, we will assume that *I_g_*/*I_s_* equals the ratio of the FITC-dextran concentrations inside and outside the microgel, i.e., the partition coefficient.

The gradually decreasing intensity ratio with the increasing molecular weight shows that there was no “cutoff” at some molecular weight above which the mesh size of the network would prohibit the probes from entering the microgels. Instead, the uniform distribution of the intensity ratio inside the microgels shows that the microgels were permeable to all investigated probes and homogeneous on length scales down to the resolution of the confocal microscopy images (~0.2 µm), consistent with their optical transparency. Little can be said about the structure of the network on shorter length scales, except that there must be continuous domains with a minimum mesh size of ca. 22 nm (the hydrodynamic diameter of the largest probe used). However, even for the smallest probe investigated (4 kDa), the concentration inside the microgels was 20–40% lower than that in the liquid outside. Since the HA concentration was less than a few weight percent in all microgels, the concentration difference remains even if one corrects for the volume occupied by the gel network. Neither is it realistic that the probe would be completely excluded from 20–40% of the microgel volume since the network in those domains would need to have a mesh size smaller than the hydrodynamic diameter of the probe (3 nm), corresponding to the length of just three disaccharide units. Rather, since FITC carries one negatively charged carboxylate group at pH 7.4 [53], we attribute a substantial part of the effect to electrostatic interactions with the negatively charged HA network. According to the model calculations underlying the theoretical swelling curves in Figure 1, the mobile monovalent anions were partly excluded from the microgels due to the Donnan effect. For example, for the microgels of batch 2.1, 2.2, and 2.3, the mobile anion concentration in the gel relative to that in a 5 mM salt solution outside was found to be 0.35, 0.30, and 0.18, respectively. The calculations are expected to overestimate the exclusion effect, in part because the model neglects ion binding to HA (assumption of uniform electrostatic potential inside the gel) and in part because the HA concentration is assumed to be equal to the average concentration in the microgel (assumption of homogeneous gel). Nevertheless, if the FITC-dextran partitioned in about the same way as a simple anion, it would clearly be excluded from the microgels to a large extent and the effect would increase with the increasing HA concentration in the gel, which is in agreement with our experimental observations (cf. Figure 4E). However, as long as each dextran molecule carried only one FITC per molecule, the effect would be the same for all molecular weight fractions. Thus, the strong molecular weight dependence observed is clearly indicative of a size exclusion effect, as expected from previous studies. In their seminal work, Laurent and co-workers [54,55] concluded that semi-dilute hyaluronic acid solutions and covalently crosslinked HA gels partly excluded proteins due to “passive” sieving attributed to the excluded volume interaction with the HA chains. Their data were in semi-quantitative agreement with a theory by Ogston et al. [56], which was based on a model of the excluded volume interaction between the spheres (protein) and a random mesh of stiff rods (HA). According to the model, the partition coefficient (K) is related to the volume fraction of the rods (∅) and the radii of the sphere ($r_{S}$) and rod ($r_{R}$) in the following way:

(5) $$K=exp\left\{-\emptyset {\left(1+\frac{r_{S}}{r_{R}}\right)}^{2}\right\}$$

**Figure 5:**
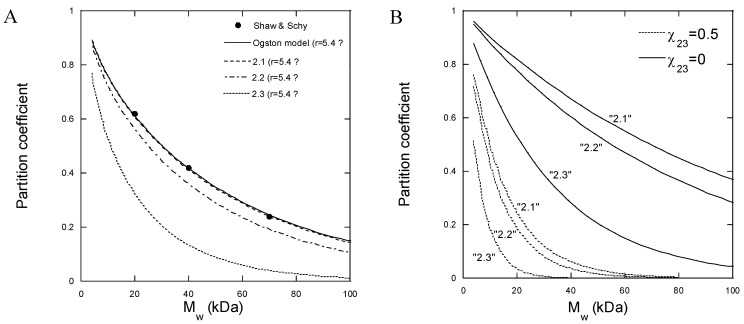
Partition coefficient of dextran between liquid solution and HA gel as a function of M_w_ of dextran, defined as the ratio of the volume fraction of dextran in the HA gel and the liquid solution. (**A**): Experimental data by Shaw and Schy [57] (points) and theoretical curves calculated from the Ogston theory [56]; Solid curve: fit to experimental data; Broken curves: Calculated with parameters relevant for batches 2.1–2.3 and $r_{R}=5$.4 Å. (**B**): Partition coefficients calculated from the Flory–Huggins/modified Flory–Rehner theories with dextran–HA interaction parameters (χ_23_) as indicated and gel model parameters for each curve taken from Table 2, as described in Table S4.

Figure 5A shows the partition coefficient for partitioning between an aqueous solution and covalently crosslinked HA gels for three FITC-dextran probes of different molecular weights, determined experimentally by Shaw and Schy [57]. The solid line is a fit of Equation (5) to the data with $r_{R}$ as a fitting parameter; ∅ was calculated from the concentration of HA in the gel (0.015 g/mL) and the partial specific volume of HA (0.65 mL/g), and $r_{S}$ was assumed to be equal to the hydrodynamic radius of the probes. We calculated $r_{S}$ from the mathematical relationship between the molecular weight and hydrodynamic radius determined from the data in Table 3 (see Table S3). Clearly, with $r_{R}=5.4$ Å, the Ogston model provides a good fit to the data. The broken lines in Figure 5A were calculated from Equation (5) with the same $r_{R}$ and with HA concentrations representative for batches 2.1, 2.2, and 2.3 in our work. The HA concentrations derived from the gel model fits in Figure 1B were 1.5, 1.8, and 3.5% (*w*/*v*), respectively. According to the model, the excluded volume effect should increase with the increasing HA concentration, which is in qualitative agreement with our measured intensity profiles in Figure 4E. However, the theoretically calculated partition coefficients are larger for the lower probe molecular weights and smaller for the higher molecular weights than observed experimentally with our microgels. Thus, the model described well the data in the narrow molecular weight range used by Shaw and Schy [57] but overestimated the dependence on the probe size for our microgels.

Since gels have properties in common with semi-dilute polymer solutions, an alternative approach to calculating partition coefficients not restricted to spheres is to apply scaling theory for linear and branched polymers in semi-dilute solutions [58,59]. The confinement experienced by a chain due to the presence of the surrounding chains in good solvents resembles that of chains confined to spherical cavities [60]. Based on the predicted free energy of perturbing the polymer chains, we found that the partition coefficients calculated from such models (data not shown) decreased with the increasing molecular weight, but the partition coefficients were much larger for the low molecular weights (*K*≈ 1) and smaller for the higher molecular weights compared to our results, which is similar to the Ogston model results. In both cases, the discrepancy at low molecular weights can be attributed to the electrostatic effect described above.

Although the importance of taking into account other types of interactions, such as electrostatic, hydrophobic, and bio-specific interactions, has been pointed out [61,62,63], the Ogston theory and related treatments [64] of excluded volume interactions are the ones most frequently applied in the literature to explain the size exclusion effects and partition coefficients [65,66,67,68]. However, for polymeric species such as dextran, the exclusion from a polymer gel can also be viewed as a “polymer incompatibility” problem, i.e., the effect behind the segregative phase separation in aqueous mixtures of two water-soluble polymers [69]. To demonstrate the potential importance of the latter, we calculated the partition coefficients based on the Flory–Huggins theory for solvent–polymer 1–polymer 2 mixtures [70] and the modified Flory–Rehner theory of gels (Equations (1)–(4)). All equations used in the calculation are provided in Table S4. The Flory–Huggins interaction parameters for dextran–water and HA–water were set to 0.5 [71] and 0.439 [52], respectively. Figure 5B shows the results of the calculations with parameters relevant for batches 2.1, 2.2, and 2.3. With no net interaction between dextran and HA ($\chi_{23}$ = 0), the partition coefficients for the low-dextran molecular weights were larger than observed experimentally. However, by introducing repulsion between the polymers by setting the interaction parameter $\chi_{23}$ = 0.5, the partition coefficients became comparable to those observed experimentally. In contrast, for the higher dextran molecular weights, the agreement between the theory and experiment was better with $\chi_{23}$ = 0. Since the theory does not take branching into account, one may speculate that the solution properties of dextran become more influenced by the branching with increasing molecular weights (see above) so that more of the chain segments are hidden inside the molecule and thus avoid contact with the HA chains. However, there are also other well-known limitations of the theory [47] that could contribute to the discrepancies. We conclude that, although the Flory–Huggins theory was not in quantitative agreement with the molecular weight dependence of the partition coefficient, the results show that polymer–polymer repulsive interactions other than excluded volume interactions may greatly influence the partition coefficient.

Looking finally at the effect of the degree of modification, the experimental data on batches 2.1–2.3 (Figure 4E) with *f* = 21% show that the partition coefficient for a given probe decreased with the increasing HA concentration in the microgel. The same conclusion holds for batches with other degrees of modification (Figure 4D,F). By neglecting the deviant batch 1.1, Figure 4A–C show that changing the degree of modification did not greatly affect the partitioning of gels prepared at the same HA concentration in the reaction mixture. This is consistent with the small variation in the number of disaccharide segments between crosslinks as determined from the gel model fits (Table 2).

To summarize, the microgels were permeable to all tested FITC-dextran probes, showing that the effective mesh size of the microgels was at least 22 nm. Partitioning from the liquid to the microgel decreased with the increasing probe molecular weights and increasing HA concentrations in the microgel but there was no significant effect of the crosslinking as such. The results suggest that the partitioning of dextran to the microgels was limited mainly by the excluded volume interactions and/or net repulsive intermolecular forces between dextran and HA in water. Those types of interactions are not consequences of the network architecture or the presence of covalent crosslinks between the HA chains. Thus, when employing dextran diffusion probes of different molecular weights to determine the “pore size” of gels, it is important to keep in mind that the probes should not, in general, be considered “inert” with respect to interactions with the gel material.

**Multiple regression analysis.** We fitted the volume ratio *V*/*V_0_* of the different gels at 1 M NaCl (Table 2) and the fluorescence intensity ratios (Table S2) to a statistical model with multiple regression analysis (MLR) (see Table S5). The results of the analysis were in agreement with the above conclusions.

## 3. Conclusions

**Figure 6:**
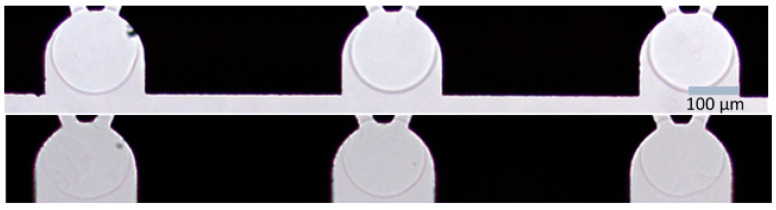
HA microgels in hydrodynamic traps on a microfluidic chip (MIS) in PB with 50 mM NaCl. Microgels 1.3 (**upper**) and 1.1 (**lower**).

The results show that it is possible to synthesize highly responsive microgels (“beads”) of diameter 100–500 µm by covalently crosslinking pre-modified linear HA in microscopic aqueous droplets created using microfluidics. The swelling responsiveness of the microgels could be tuned in a reproducible way by varying the fraction of ethylacrylamide-modified disaccharide units in the HA chains and the HA concentration in the gelling solution. However, the number of crosslinks per chain was lower than the number of modifications and the yield of the crosslinking reaction decreased with the increasing degree of the modification. For all microgel compositions investigated, the networks were fully permeable to the FITC-dextran diffusion probes of molecular weights up to at least 250 kDa, showing that the HA network was homogeneous on mesoscopic to macroscopic length scales and that the limiting mesh size was larger than 22 nm. Partitioning from the solution to the microgel decreased with the increasing molecular weight of the FITC-dextrans. The results are in qualitative agreement with the expectations for homogeneous microgels giving rise to a size exclusion effect deriving from the excluded volume and net repulsive intermolecular forces between HA and dextran in the presence of water. Overall, the results show that the microgels, in particular the ones with the lowest degrees of modification of the HA, have properties suitable in applications directed towards quantifying the strength of interactions between HA and proteins, peptides, and other macromolecules. One such application with the potential to be used as a screening tool in the development of protein and peptide drugs intended for subcutaneous administration [31] is a novel miniaturized in vitro method based on microfluidics that was described in a recent paper from our lab [30]. The sensitivity of the method increases with the increasing responsivity of the gel networks. However, increasing the responsivity by lowering the degree of modification and amount of HA too much will lead to two problems. First of all, it may become problematic to determine the size of the microgels due to contrast limitations when using optical microscopy. In Figure 6, microgels 1.1 and 1.3 are shown in the traps of the MIS. Both batches of microgels can still be captured with optical microscopy and the size can be measured, but the contour of the microgels becomes less distinct the more the degree of modification and amount of HA in microgels are lowered until it is impossible to accurately measure the volume of the microgels. Furthermore, at some point, the stability of the microgels becomes too low, which could make them break because of the mechanical stress applied when loading the microgels onto the chip or rapidly degrade chemically because of the low number of crosslinks between the polyelectrolyte chains.

## 4. Materials and Methods

**Materials.** Polydimethylsiloxane (PDMS) DowSyl Sylgard™ 184 (including elastomer base and curing agent) from GA Lindberg ChemTech AB (Stockholm, Sweden), Picosurf™ 5% in Novec™ 7500 from Sphere Fluidics (Cambridge, UK), Novec™ 7500 (>99%) from 3M (Saint Paul, MN, USA), sodium hyaluronate (100–300 kDa) from Contipro a.s (Dolní Dobrouč, Czech), N-(2-aminoethyl) acrylamide hydrochloride (AEA) from abcr GmbH (Karlsruhe, Germany), 2-propanol (ACS reagent) from Merck KGaA (Darmstadt, Germany), ethanol (99.7%) from Solveco (Rosersberg, Sweden), (N-(3-dimethylaminopropoyl)-N′-ethylcarbodiimide hydrochloride (EDC) from Acros Organics (Geel, Belgium), and mr-Dev 600 from Micro Resist Technology GmbH (Berlin, Germany), were all used as received. Spectra/Por^®^ 6 RC-membrane (3.5 kDa cutoff) was purchased from SpectrumLabs (Rancho Dominguez, CA, USA), and SUEX photoresist film was from DJ MicroLaminates (Sudbury, MA, USA).

The following were all purchased from Sigma Aldric (Sent Louis, MO, USA), SE: Sigmacote^®^, sodium chloride (≥99%), fluorescein isothiocyanate-dextran (FITC- Dextran 4, 10, 40, 70, and 250 kDa), HOBt (1-hydroxybenzotriazole hydrate, ≥97.0%), phosphate monobasic (ReagentPlus ≥99%), sodium phosphate dibasic (ReagentPlus ≥99%), acetonitrile (anhydrous 99.8%), lithium phenyl-2,4,6-trimethylbenzoylphosphinate (LAP, 900889), sterile syringe filters (5 µm, Merck Millipore, Burlington, MA, USA), 1H, 1H, 2H, 2H-perfluoro-1-octanol (≥97%), and sodium hydroxide (≥97%).

Photoresist S1813, Microposit™ 351 developer, H_3_PO_4_, CO_3_COOH, and HNO_3_ were all lab grade and provided by the Ångström Microstructure laboratory, Uppsala, Sweden.

**Fabrication of microfluidic chip for droplet production**. The exact procedure for the manufacturing of the microfluidic chips for droplet production (MDP) used in this work has been described in detail in a previous work by Wanselius et al. [30]. The fabrication was carried out using standard soft lithography techniques [72]. In brief, the mold for the microfluidic chip was fabricated by laminating a silicon wafer with SUEX photoresist film. The laminated wafer was exposed to UV light at a wavelength of 365 nm, crosslinking the SUEX photoresist to the silicon wafer according to a template. The non-crosslinked laminate was removed using the development solution mr-dev 600.

The microfluidic chips casted using the mold were made of PDMS (Sylgard 184). Casted PDMS structures were covalently bound to glass slides. The MDP were treated with Sigmacote^®^ immediately before use to make the channels hydrophobic. For a schematic picture of the MDP, see Wanselius et al. [30].

**Synthesis of hyaluronic acid–ethylacrylamide and production of microspheres.** The functionalization of HA was based on a protocol by Shi et al. [73]. In brief, 400 mg (1 mmol) sodium hyaluronate (Mw 130–300 kDa) was dissolved by stirring overnight in milli-Q water at a concentration of 8 mg/mL. After adding the crosslinker (N-(2-aminoethyl)acrylamide hydrochloride) in amounts corresponding to targeted degrees of modification (Table 1), the reaction mixture was kept in the dark. HOBt was separately dissolved upon gentle heating in a 1:1 (*v*/*v*) mixture of water/acetonitrile and cooled to room temperature before adding it to the reaction mixture. The pH was adjusted to 6.0 by adding 1 M NaOH. The reaction was started by the addition of EDC (≥99%) and allowed to proceed for 24 h under stirring. After that, the ethylacrylamide-modified hyaluronic acid solution was dialyzed against dilute HCl pH 3.5 containing 100 mM NaCl for 24 h, after which the dialysis was continued against NaCl-free HCl solution (pH 3.5) for two days and milli-Q water for another two days. After adjusting the pH to 7 with 1 M NaOH, the solution was filtered (5 µm), then frozen in liquid nitrogen, and finally freeze-dried for 5 days, resulting in a white, soft product.

The degree of modification was determined by ^1^H NMR using the resonance at 1.9 ppm (corresponding to protons of the N-acetyl group of hyaluronic acid) for normalization. Proton resonances at 5.6 and 6.1 ppm confirmed the presence of the introduced ethylacrylamide groups, respectively. ^1^H NMR spectra and more details about the amounts of material used for synthesis can be found in the Supplementary Materials (Figures S12–S15, Tables S3–S6)

Crosslinked HA microgels were fabricated using the MDP, a droplet-making chip with a flow-focusing design, the principle of which has been described in detail elsewhere [30]. Freeze-dried HA–ethylacrylamide with different degrees of modification were dissolved in DI water with 0.1% (*w*/*w*) to a concentration of either 1.5, 2, or 3% (*w*/*w*). Novec™ 7500 with 0.5% (*v*/*v*) Picosurf™ was used as the continuous oil phase. The two liquids were connected to the MDP and flow rates were set to 5 µL/min (aqueous phase) and 120 µL/min (oil phase) using an OBK Mlll+ pressure pump from Elveflow (Elvesys, Paris, France). The generated w/o emulsion droplets were collected and crosslinked with UV light at a wavelength of 365 nm and irradiation energy of 1000 µJ/cm^2^ for 10 min using a UVP crosslinker CL-1000 (Analytikjena Jena GmbH, Jena, Germany). The emulsion was broken by adding 1H, 1H, 2H, 2H-perfluoro-1-octanol, and the (aq) phase was transferred into PB and stored at 4 °C.

**Fabrication of microfluidic chip for interaction studies.** The swelling response studies were performed on single microgels confined to hydrodynamic traps on a custom-designed microfluidics chip. The setup, referred to as microfluidic chips for interaction studies (MIS), has been evaluated and described in detail elsewhere [30]. The chip comprised a 200 µm-thick silicon wafer sandwiched between two glass wafers consisting of a bottom 1 mm-thick wafer and a top 0.5 mm-thick wafer. The chip was produced using standard etching techniques as follows.

The silicon wafer was first cleaned by the standard RCA clean (Radio Corporation of America), followed by an extra cleaning step of exposure to HNO_3_ at 75 °C for 15 min. The silicon was then bonded to the 1 mm glass-bottom wafer overnight through anodic bonding (1200 V at 380 °C). A hard mask of aluminum (Al) with a thickness of 500 nm was deposited onto the silicon wafer through sputtering (Power 1000 W, duration 2 min) using a Von Ardenne Magnetron model CS730S. The wafers were then left to dehydrate for 5 min at 200 °C.

A 1 µm-thick layer of photoresist S1813 was spin-coated onto the Al mask and soft-baked for 2 min at 110 °C. After cooling off for a few hours, hard contact UV lithography was used for design pattering (4 s exposure) followed by the removal of non-crosslinked photoresist by exposure to developer Microposit 351 (45 s exposure). The wafers were then hard-baked for 5 min at 120 °C before dry-etching through the Al hard mask for 65 s, followed by dry-etching through the silicon (20 s/cycle, 215 cycles). Both processes were performed using an ICP-RIE from Plasma-Therm. After the dry-etching, the wafers were washed with acetone and 2-propanol before the remaining Al was removed by wet-etching (Ratio H_3_PO_4_:CO_3_COOH:HNO_3_ 29:5:1, at 40 °C). Holes for inlets and outlets were cut in the 0.5 mm glass wafer using a laser cutter from Östling Marking systems (Solingen, Germany). The 0.5 mm glass wafer and silicon wafer were then washed with HNO_3_ at 75 °C for 15 min before anodic bonding between the two overnight (1200 V at 380 °C). The sandwiched bonded wafers were then diced into individual MIS. Finally, the PDMS was cast into 3 mm-thick films, cut into squares, and used as connectors for tubing into the glass chip. Holes 0.75 mm in diameter were punched through the PDMS squares and the squares were covalently bonded to the glass using a Harrick Plasma cleaner PDC-32 G. Exposure time to plasma was 30 s, followed by 1 h in the oven at 70 °C. A schematic illustration of the MIS has been presented elsewhere [30].

**Determination of microgel volume.** An Olympus BX51 microscope equipped with an UMPlanFI 5× lens and an Olympus DP73 digital camera was used for capturing the images of the microgels. The imaging software cellSens Dimension version 1.7.1 from the Olympus Corporation was used for determining the diameters of the microgels; microgel volumes were then calculated from these diameters. The experiments were carried out in PB (phosphate-buffered media containing 3.5 mM sodium phosphate monobasic and 1.5 mM sodium phosphate dibasic) containing various concentrations of NaCl. Small amounts of HCL were used to adjust the pH to 7.4.

**Ionic strength response measurements.** The MIS was used to investigate the microgel volume responses upon exposure to solutions with different NaCl concentrations. Two tubes (Masterflex EW-06417-11) with an ID of 300 µm were connected to the microfluidic chip via flow sensors. A stock solution with 1 M NaCl in PB was connected to one of the chip inlets and the PB with no added NaCl was connected to the other. The two solutions were perfused through the microfluidic chip using pressure pump (OBK Mlll+) flow sensors all from Elveflow (Elvesys, Paris, France), and the flow rates were controlled with the Elveflow smart interface software. Different ionic strengths could be acquired without changing solutions by modifying the flow rates of the two connected solutions. Before the start of each experiment, but after the microfluidic chip was filled with the PB, the microgels were manually loaded into the traps of the chip using a syringe via a third tube attached to the chip. When the microgels were trapped, the flow rates were modified to obtain the desired concentration of NaCl perfusing the trapped microgels. The flows to achieve each concentration were determined using the total flow rate of 200 µL/min for all experiments and then calculating the proportions needed to dilute a 1 M NaCl stock solution to the desired concentration in the chip (e.g., a concentration of 100 mM is given by a ratio of 1:9 or 20:180 µL/min stem solution:buffer). The concentrations varied between zero and 1 M NaCl.

**Dextran partitioning and permeability test.** HA microgels were left to equilibrate for a minimum of seven days in 2 mg/mL aqueous solutions of FITC-dextran conjugates with molecular weights ranging from 4 to 250 kDa. The microgels and solution were examined with a Zeiss LSM 700 confocal laser scanning microscope at an excitation wavelength of 488 nm/emission wavelength of 520 nm. The fluorescence intensity difference between the microgels and the surrounding solution was determined using ImageJ software. An intensity ratio of *I_g_*/*I_s_*, where *I_g_* denotes the fluorescence intensity of the gel and *I_s_* the intensity of the surrounding solution, was calculated to acquire a relative measurement of the concentration of FITC-dextran in the gels. The lowest ratio acquired from each experiment (which corresponds to the layer closest to the middle of the microgel) was used to compare the experiments with different FITC-dextran sizes or HA microgels.

**Design of experiment.** The study was performed as a full factorial experimental design with 2 factors: ethylacrylamide modification degree of HA and amount of modified HA in aqueous solution during gel production. The degree of modification was set at three levels, 13, 21, and 33%, and the amount of modified HA was at 1.5, 2, and 3% *w*/*w*. The results were evaluated with the software MODDE Pro 12 (Umetrics MKS AB, Umeå, Sweden) and the model was fitted with multiple linear regression analysis (MLR).

## Figures and Tables

**Table 1:** Equivalent ratios of precursor materials used for ethylacrylamide modification of HA, targeted degree of modification ($f_{0}$), and degree of modification experimentally determined by ^1^H NMR (*f*).

Material	Sodium Hyaluronate	N-(2-Aminoethyl) Acrylamide Hydrochloride	HOBt	EDC	$f_{0}\;\;(\%)$	*F* (%)
Molar equivalent ratios	1	0.74	0.95	1.42	70	33
	1	0.66	0.94	1.42	88	21
	1	0.55	0.94	1.40	55	21
	1	0.54	0.94	1.42	50 ^1^	13

^1^ Shortened reaction time to 8 h instead of 24 h.

**Table 2:** Ethylacrylamide modification degree of HA and amount (% *w*/*w*) of HA in the aqueous solution used for the production of the 12 different batches of microgels presented in this work, and the results from the swelling response experiments.

Microgel	Degree of Modification (*f*) (%)	[HA] in Solution during Microgel Production % (*w*/*w*)	*V*/*V_0_* at 1 M NaCl	Responsivity (*V_0_*/*V*)	*M* ^1^	Number of Disaccharide Units between Cross-Links ^2^
1.1	13	1.5	0.32 (±0.015)	3.1	0.86	13
1.2	13	2	0.33 (±0.017)	3.0	0.92	14
1.3	13	3	0.36 (±0.011)	2.8	0.92	14
2.1	21	1.5	0.31 (±0.012)	3.2	1.3	20
2.2	21	2	0.33 (±0.006)	3.0	1.3	20
2.3	21	3	0.42 (±0.007)	2.4	0.80	12
3.1	21	1.5	0.32 (±0.008)	3.1	0.80	12
3.2	21	2	0.35 (±0.010)	2.9	0.80	12
3.3	21	3	0.41 (±0.013)	2.4	0.75	11
4.1	33	1.5	0.36 (±0.016)	2.8	1.1	16
4.2	33	2	0.40 (±0.021)	2.5	0.90	14
4.3	33	3	0.48 (±0.014)	2.1	0.74	11

^1^ Number of statistical segments between crosslinks obtained from the fit of gel model, Equations (1)–(4), to the experimental data.^2^ Calculated from *M* assuming 15 disaccharide units per statistical segment.

**Table 3:** Molecular weights and hydrodynamic radii of FITC-dextran probes.

M_w_ (kDa)	Hydrodynamic Radius (nm)
4	1.4
10	2.3
40	4.5
70	6.0
150	8.5
250	(11) ^a^

^a^ Estimated from extrapolation.

## Data Availability

Not Applicable.

## Supplementary Materials

The following supporting information can be downloaded at: https://www.mdpi.com/article/10.3390/gels8090588/s1. S1. HA microgel volume response to NaCl full dataset. S2. Intensity ratios of FITC-dextran full data set, tables, and images. S3. Relationship between the hydrodynamic radius and molecular weight for dextrans. S4. Calculation of partition coefficient based on the Flory–Huggins theory. S5. Multiple regression analysis of acquired *V*/*V_0_* and *Ig*/*Is* results. S6. ^1^HNMR characterization of ethylacrylamide-modified hyaluronic acid, details about amounts used during functionalization.
