# Supplementary material for: Responsive Hyaluronic Acid–Ethylacrylamide Microgels Fabricated Using Microfluidics Technique
Source: Gels. 2022 Sep 15;8(9):588. doi: 10.3390/gels8090588 (PMC9498840; doi:10.3390/gels8090588)
Supplement: Supplementary file 1 [file gels-08-00588-s001.zip › gels-1899197-supplementary.pdf]

# Supplementary Materials

# Responsive Hyaluronic Acid-Ethylacrylamide Microgels Fabricated Using Microfluidics Technique

*Marcus Wanselius[a], Agnes Rodler[a], Sean S. Searle[a], Susanna Abrahmsén-Alami[b], Per Hansson[a]**

a) Department of Medicinal Chemistry, Uppsala University, SE-751 23, Uppsala, Sweden

b) Innovation Strategies & External Liaison. Pharmaceutical Technology & Development, Operations, AstraZeneca, SE-43183, Gothenburg, Sweden

## S1. HA microgel response to NaCl

Below we present the volume change of all 12 different batches at exposure to 10 different NaCl concentrations.

**Table S1.** Volume ratio ($V_{end}/V_0$) of microgels exposed to 0-1 M NaCl in PBS 5 mM pH 7.4, flow rate 200 µl/min. $V_0$ being the volume of the microgels in PBS 5 mM pH 7.4. Values are the mean value of 8 different gels in the same experiment.

| NaCl concentration (mM) 0 | 10 | 25 | 50 | 75 | 100 | 125 | 150 | 200 | 1000 |
|---|---|---|---|---|---|---|---|---|---|

| Microgel | Volume ratio ($V/V_0$) | | | | | | | | | |
|---|---|---|---|---|---|---|---|---|---|---|
| 1.1 | 1 (±0) | 0.885 (±0.015) | 0.700 (±0.032) | 0.595 (±0.020) | 0.531 (±0.020) | 0.494 (±0.023) | 0.463 (±0.018) | 0.447 (±0.021) | 0.416 (±0.021) | 0.321 (±0.015) |
| 1.2 | 1 (±0) | 0.864 (±0.019) | 0.710 (±0.027) | 0.586 (±0.028) | 0.532 (±0.018) | 0.495 (±0.024) | 0.474 (±0.021) | 0.443 (±0.016) | 0.423 (±0.020) | 0.330 (±0.017) |
| 1.3 | 1 (±0) | 0.889 (±0.028) | 0.727 (±0.026) | 0.624 (±0.019) | 0.563 (±0.017) | 0.535 (±0.020) | 0.512 (±0.013) | 0.488 (±0.012) | 0.462 (±0.014) | 0.359 (±0.011) |
| 2.1 | 1 (±0) | 0.790 (±0.014) | 0.596 (±0.028) | 0.493 (±0.023) | 0.450 (±0.020) | 0.426 (±0.024) | 0.409 (±0.021) | 0.391 (±0.019) | 0.371 (±0.019) | 0.310 (±0.012) |
| 2.2 | 1 (±0) | 0.794 (±0.014) | 0.611 (±0.014) | 0.504 (±0.014) | 0.462 (±0.013) | 0.436 (±0.011) | 0.420 (±0.010) | 0.403 (±0.010) | 0.385 (±0.012) | 0.328 (±0.006) |
| 2.3 | 1 (±0) | 0.887 (±0.022) | 0.730 (±0.012) | 0.631 (±0.020) | 0.592 (±0.016) | 0.561 (±0.016) | 0.538 (±0.017) | 0.520 (±0.008) | 0.498 (±0.009) | 0.421 (±0.007) |
| 3.1 | 1 (±0) | 0.896 (±0.020) | 0.738 (±0.019) | 0.616 (±0.014) | 0.559 (±0.020) | 0.516 (±0.018) | 0.488 (±0.018) | 0.464 (±0.018) | 0.429 (±0.015) | 0.323 (±0.008) |
| 3.2 | 1 (±0) | 0.916 (±0.023) | 0.748 (±0.017) | 0.627 (±0.008) | 0.571 (±0.011) | 0.537 (±0.013) | 0.506 (±0.010) | 0.487 (±0.007) | 0.453 (±0.013) | 0.349 (±0.010) |
| 3.3 | 1 (±0) | 0.919 (±0.018) | 0.769 (±0.018) | 0.677 (±0.014) | 0.629 (±0.016) | 0.590 (±0.014) | 0.566 (±0.011) | 0.545 (±0.012) | 0.510 (±0.010) | 0.406 (±0.013) |
| 4.1 | 1 (±0) | 0.808 (±0.019) | 0.626 (±0.023) | 0.529 (±0.023) | 0.479 (±0.031) | 0.465 (±0.026) | 0.450 (±0.027) | 0.426 (±0.018) | 0.401 (±0.016) | 0.355 (±0.016) |
| 4.2 | 1 (±0) | 0.841 (±0.020) | 0.668 (±0.015) | 0.582 (±0.009) | 0.537 (±0.009) | 0.509 (±0.010) | 0.493 (±0.015) | 0.472 (±0.014) | 0.455 (±0.018) | 0.402 (±0.021) |
| 4.3 | 1 (±0) | 0.875 (±0.014) | 0.744 (±0.022) | 0.651 (±0.017) | 0.615 (±0.021) | 0.593 (±0.019) | 0.575 (±0.021) | 0.560 (±0.019) | 0.541 (±0.018) | 0.484 (±0.014) |

## S2. Fluorescence intensity data from FITC-dextran probe penetration experiments

In Table S2 the intensity ratios between fluorescence inside and outside microgels exposed to the fluorescence probe FITC-dextran of different sizes for 7 days minimum. A high ratio indicates to a high penetration of the probes.

**Table S2.** Intensity ratio of fluorescence inside and outside different microgels ($I_g/I_s$), when in equilibrium with solutions of different sized FITC-Dextran. Values are mean values of 9 microgels. Degree of modification of HA and amount of HA in solution during gel production for each batch are also presented here.

| Microgels | Ethylacrylamide modification degree of HA (*f*) | Amount of HA in aq solution during microgel production (w/w) | FITC-Dextran 4 kDa | FITC-Dextran 10 kDa | FITC-Dextran 40 kDa | FITC-Dextran 70 kDa | FITC-Dextran 250 kDa |
|---|---|---|---|---|---|---|---|
| 1.1 | 30% | 1.5 % | 0.792 (±0.016) | 0.753 (±0.016) | 0.721 (±0.023) | 0.528 (±0.017) | 0.451 (±0.019) |
| 1.2 | 30% | 2 % | 0.637 (±0.009) | 0.573 (±0.012) | 0.435 (±0.021) | 0.315 (±0.009) | 0.315 (±0.006) |
| 1.3 | 30% | 3 % | 0.603 (±0.031) | 0.504 (±0.023) | 0.367 (±0.007) | 0.259 (±0.016) | 0.284 (±0.010) |
| 2.1 | 46 % | 1.5 % | 0.748 (±0.013) | 0.627 (±0.020) | 0.544 (±0.015) | 0.423 (±0.017) | 0.410 (±0.019) |
| 2.2 | 46 % | 2 % | 0.635 (±0.019) | 0.586 (±0.032) | 0.481 (±0.035) | 0.298 (±0.009) | 0.318 (±0.010) |
| 2.3 | 46 % | 3 % | 0.585 (±0.024) | 0.352 (±0.023) | 0.298 (±0.013) | 0.271 (±0.028) | 0.275 (±0.006) |
| 4.1 | 68 % | 1.5 % | 0.690 (±0.017) | 0.589 (±0.019) | 0.531 (±0.018) | 0.434 (±0.014) | 0.439 (±0.004) |
| 4.2 | 68 % | 2 % | 0.596 (±0.021) | 0.529 (±0.005) | 0.443 (±0.012) | 0.334 (±0.004) | 0.329 (±0.008) |
| 4.3 | 68 % | 3 % | 0.514 (±0.008) | 0.413 (±0.007) | 0.375 (±0.004) | 0.317 (±0.007) | 0.311 (±0.010) |

Below are confocal images of 9 different batches of gels exposed to 5 different sized FITC-Dextran Probes (4, 10, 40, 70 and 250 kDa).

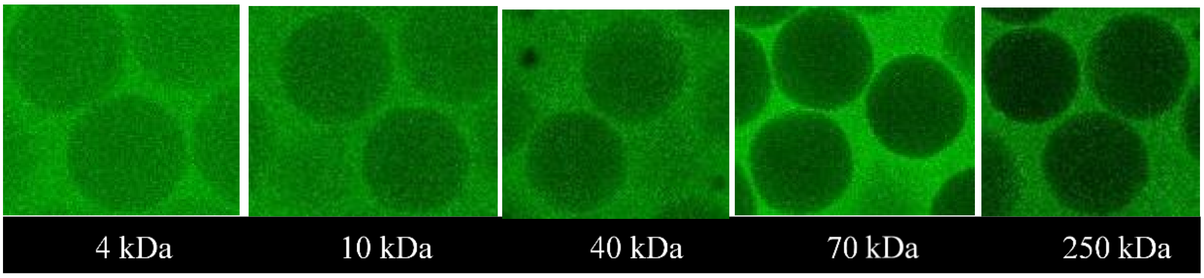


**Figure S1**. CLSM false-color images of central focal plane of microgels (1.1) in equilibrium with aqueous solutions of different sized FITC-Dextran. Size of gels approximately 250 µm in diameter.

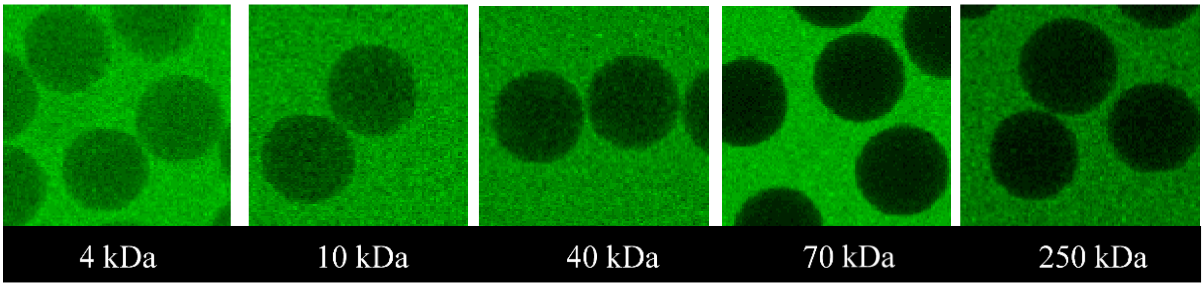


**Figure S2**. CLSM false-color images of central focal plane of microgels (1.2) in equilibrium with aqueous solutions of different sized FITC-Dextran. Size of gels approximately 200 µm in diameter.

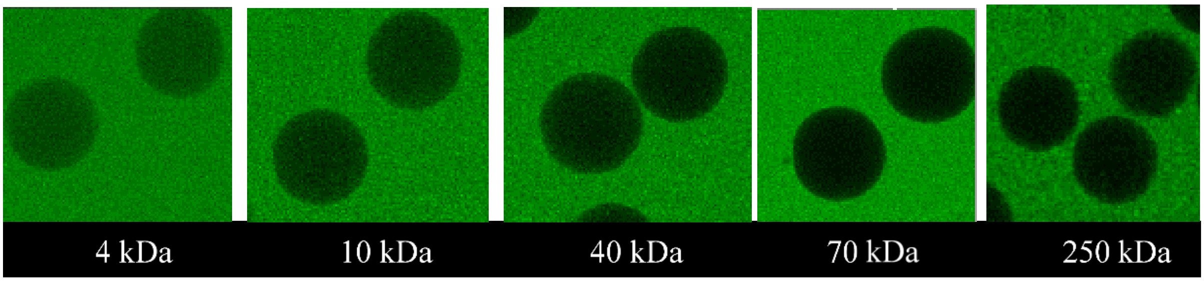


**Figure S3**. CLSM false-color images of central focal plane of microgels (1.3) in equilibrium with aqueous solutions of different sized FITC-Dextran. Size of gels approximately 150 µm in diameter.

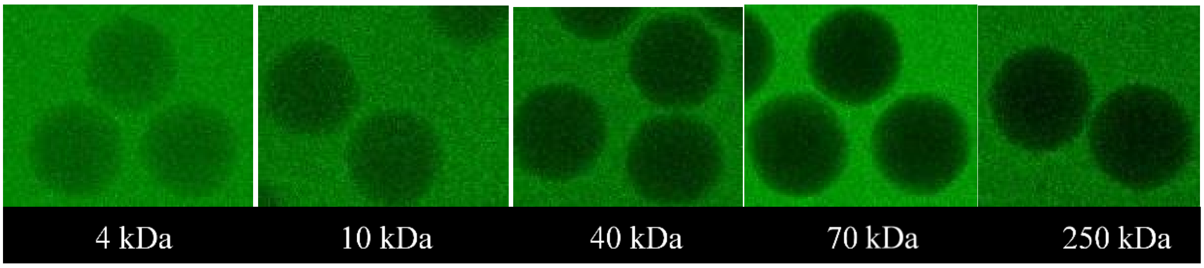


**Figure S4**. CLSM false-color images of central focal plane of microgels (2.1) in equilibrium with aqueous solutions of different sized FITC-Dextran. Size of gels approximately 150 µm in diameter.

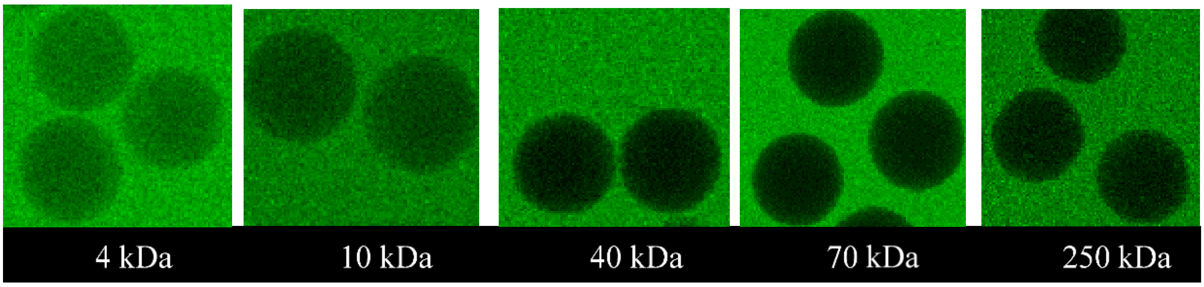


**Figure S5**. CLSM false-color images of central focal plane of microgels (2.2) in equilibrium with aqueous solutions of different sized FITC-Dextran. Size of gels approximately 150 µm in diameter.

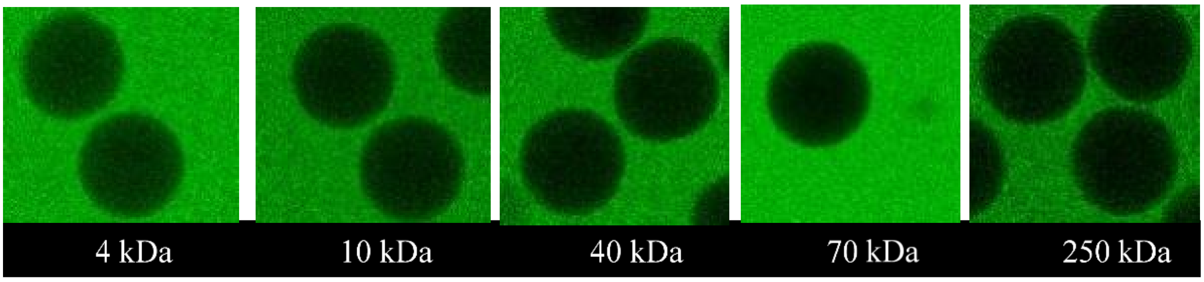


**Figure S6**. CLSM false-color images of central focal plane of microgels (2.3) in equilibrium with aqueous solutions of different sized FITC-Dextran. Size of gels approximately 150 µm in diameter.

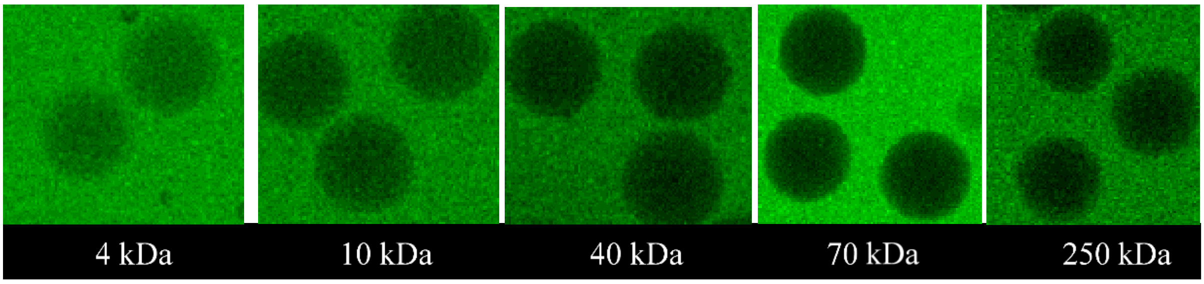


**Figure S7**. CLSM false-color images of central focal plane of microgels (4.1) in equilibrium with aqueous solutions of different sized FITC-Dextran. Size of gels approximately 150 µm in diameter.

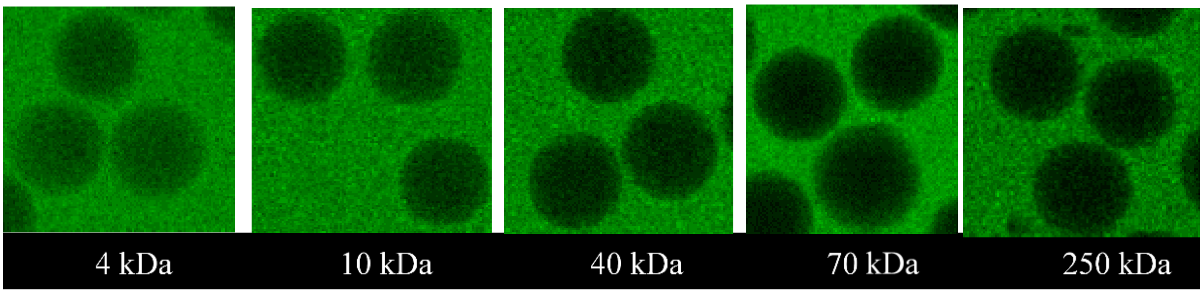


**Figure S8**. CLSM false-color images of central focal plane of microgels (4.2) in equilibrium with aqueous solutions of different sized FITC-Dextran. Size of gels approximately 150 µm in diameter.

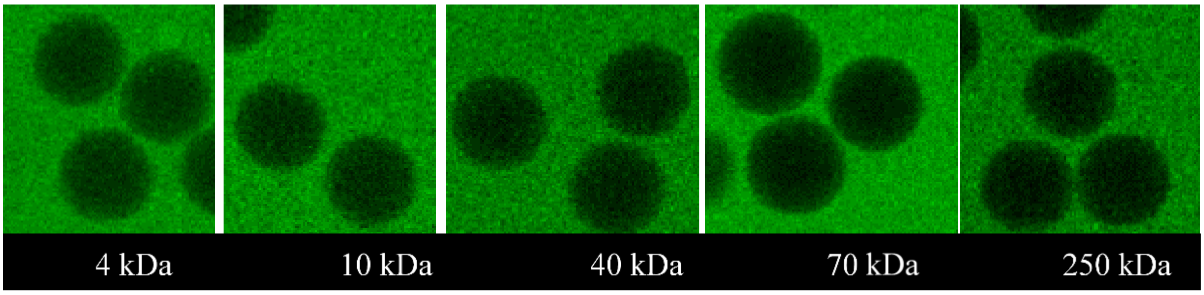


**Figure S9.** CLSM false-color images of central focal plane of microgels (4.3) in equilibrium with aqueous solutions of different sized FITC-Dextran. Size of gels approximately 150 µm in diameter.

## S3. Relationship between hydrodynamic radius and molecular weight for dextrans

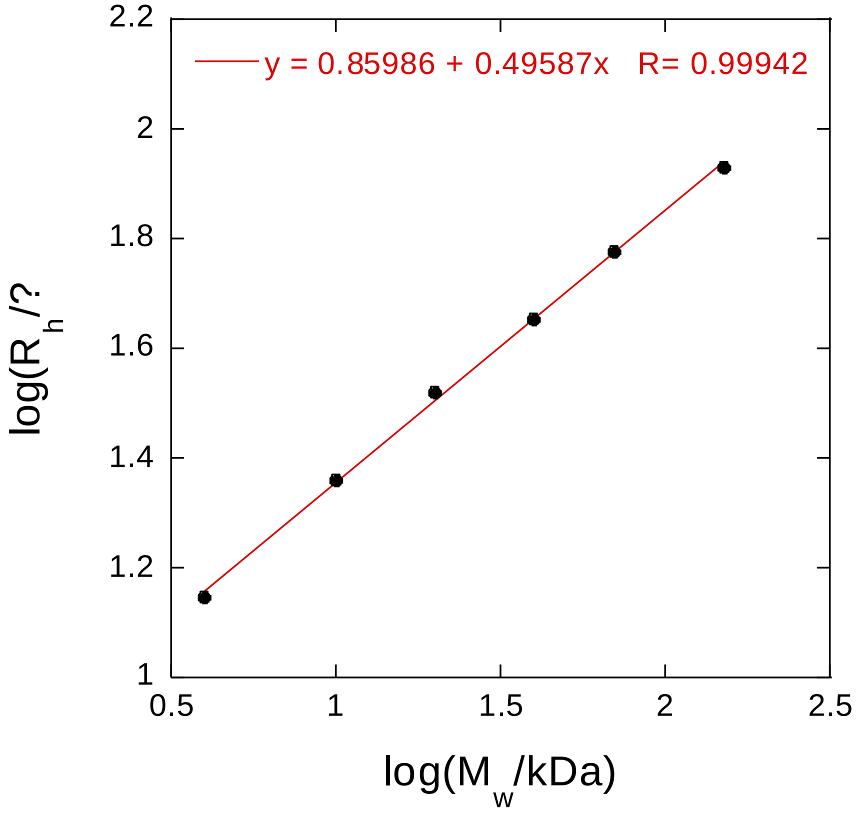


**Figure S10.** Log – log **r**elationship between the hydrodynamic radius and molecular weight for dextran diffusion probes. Data points from Table 3 in the Article. Line: Result from linear regression analysis; the resulting relationship.

## S4. Calculation of partition coefficient based on the Flory-Huggins theory

The theory is based on a lattice model of a solution of $N_1$ solvent molecules of volume $v_1$, $N_2$ molecules of one polymer of volume $p_2v_1$, and $N_3$ molecules of another polymer of volume $p_3v_1$, where $v_1$ is the volume of one site in the lattice and $p_i$ is the number of sites occupied by one molecule of species $i$ ($p_1 \equiv 1$).

The free energy of mixing is:

$$\frac{\Delta A_{mix}}{kT} = \sum_i N_i \, ln\emptyset_i + \frac{1}{2}\sum_i \sum_j \chi_{ij} N_i p_i \emptyset_j \quad \text{(S:1)}$$

where $kT$ is the thermal energy and $\chi_{ij}$ is the Flory-Huggins interaction parameter. The volume fractions are:

$$\emptyset_i = \frac{N_i p_i}{\sum_i N_i p_i} \quad \text{(S:2)}$$

The chemical potential of component $i$ is obtained by taking the derivative of $A_{mix}$ with respect $N_i$ keeping $N_{j\neq i}$ constant. The result is:

$$\frac{\Delta\mu_1}{kT} = ln\emptyset_1 + 1 - \emptyset_1 - \frac{\emptyset_2}{p_2} - \frac{\emptyset_3}{p_3} + (\chi_{12}\emptyset_2 + \chi_{13}\emptyset_3)(\emptyset_2 + \emptyset_3) - \chi_{23}\emptyset_2\emptyset_3 \quad \text{(S:3)}$$

$$\frac{\Delta\mu_2}{kT} = ln\emptyset_2 + 1 - \emptyset_2 - p_2\emptyset_1 - \frac{p_2}{p_3}\emptyset_3 + p_2(\chi_{12}\emptyset_1 + \chi_{23}\emptyset_3)(\emptyset_1 + \emptyset_3) - p_2\chi_{13}\emptyset_1\emptyset_3 \quad \text{(S:4)}$$

$$\frac{\Delta\mu_3}{kT} = ln\emptyset_3 + 1 - \emptyset_3 - p_3\emptyset_1 - \frac{p_3}{p_2}\emptyset_2 + p_3(\chi_{13}\emptyset_1 + \chi_{23}\emptyset_2)(\emptyset_1 + \emptyset_2) - p_3\chi_{12}\emptyset_1\emptyset_2 \quad \text{(S:5)}$$

In a liquid solution of component 1 and 3 the chemical potentials are:

$$\frac{\Delta\mu_1^{liq}}{kT} = ln\emptyset_1 + 1 - \emptyset_1 - \frac{\emptyset_3}{p_3} + \chi_{13}\emptyset_3^2 \quad \text{(S:6)}$$

$$\frac{\Delta\mu_3^{liq}}{kT} = ln\emptyset_3 + 1 - \emptyset_3 - p_3\emptyset_1 - \chi_{13}\emptyset_1^2 \quad \text{(S:7)}$$

Let the chains of component 2 be covalently cross-linked to form a gel network. Since the chains lack translational entropy we set $N_2 = 0$ in the first sum in eq. (S:1). Furthermore, as long as no phase transformations in the gel takes place, only the components 1 and 3 are allowed to distribute between different phases in the system. The chemical potentials of component 1 and 3 are:

$$\frac{\Delta\mu_1^{gel}}{kT} = ln\emptyset_1 + 1 - \emptyset_1 - \frac{\emptyset_3}{p_3} + (\chi_{12}\emptyset_2 + \chi_{13}\emptyset_3)(\emptyset_2 + \emptyset_3) - \chi_{23}\emptyset_2\emptyset_3 - \frac{v_1\Delta\Pi_{ion}}{kT} - \frac{v_1\Pi_{def}}{kT}$$

(S:8)

$$\frac{\Delta\mu_3^{gel}}{kT} = ln\emptyset_3 + 1 - \emptyset_3 - p_3\emptyset_1 + p_3(\chi_{13}\emptyset_1 + \chi_{23}\emptyset_2)(\emptyset_1 + \emptyset_2) - p_3\chi_{12}\emptyset_1\emptyset_2 - \frac{p_3 v_1\Pi_{def}}{kT}$$

(S:9)

The last term in both expressions is the contribution from the elastic deformation free energy of the network, where $\Pi_{def}$ is defined in eq. (4) in the Article. To account for the fact that the gel network is charged we have included in eq. (S:8) the contribution to the chemical potential from the osmotic pressure due to the monovalent ions in the system, where $\Delta\Pi_{ion}$ is defined in eq. (3) in the Article. In doing so we have lumped the entire effect from the electrostatics into $\Delta\mu_1^{gel}$ and therefore omitted the contribution from the ions in the expression for $\Delta\mu_1^{liq}$. Since the concentration of water molecules in both solution and gel is much larger than the ion concentration we have neglected, for simplicity, the corresponding contributions to the chemical potential of component 3. The sufficient condition for phase equilibrium is: $\{\Delta\mu_1^{liq} = \Delta\mu_1^{gel}; \Delta\mu_3^{liq} = \Delta\mu_3^{gel}\}$.

## S5. Multiple regression analysis.

We fitted the volume ratio $V/V_0$ of the different gels at 1 M NaCl (Table 2 in Article) and the fluorescence intensity ratios (Table S2) to a statistical model with multiple regression analysis (MLR). The associated model coefficients are presented in Fig. S11. The summary of fit (Fig. S11A) gives a measurement of how well the model predicted the actual experimental results (R2) and an estimation of how precise future predictions with the model will be (Q2). Both R2 and Q2 indicate that the MLR model describes the results well, especially the $V/V_0$ and fluorescence intensity ratios ($I_g/I_s$) at exposure to 4 kDa or 250 kDa FITC-dextran, for which

the R2 values were all over 0.9 and Q2 values around 0.89. The least accurate model was $I_g/I_s$ at exposure to 40 kDa FITC-dextran where the R2 (0.72) and Q2 (0.52) still indicate a significant model. The coefficient plot (Fig. S11B) and response contour plot (Fig. S11C) give further information about how the two factors individually affect the responsivity and penetration of FITC-dextran. $V/V_0$ and $I_g/I_s$ at exposure to 4 kDa or 10 kDa FITC-dextran are dependent on both the degree of modification and the amount of HA during gel production. Both factors are statistically significant. However, the strongest effect is seen for changes in the amount of HA during gel production. When the gels are exposed to larger FITC-dextran probes (40, 70, 250 kDa), there is only a significant effect of HA during gel production on $I_g/I_s$. This MLR analysis is in accordance with the qualitative conclusions drawn in this work.

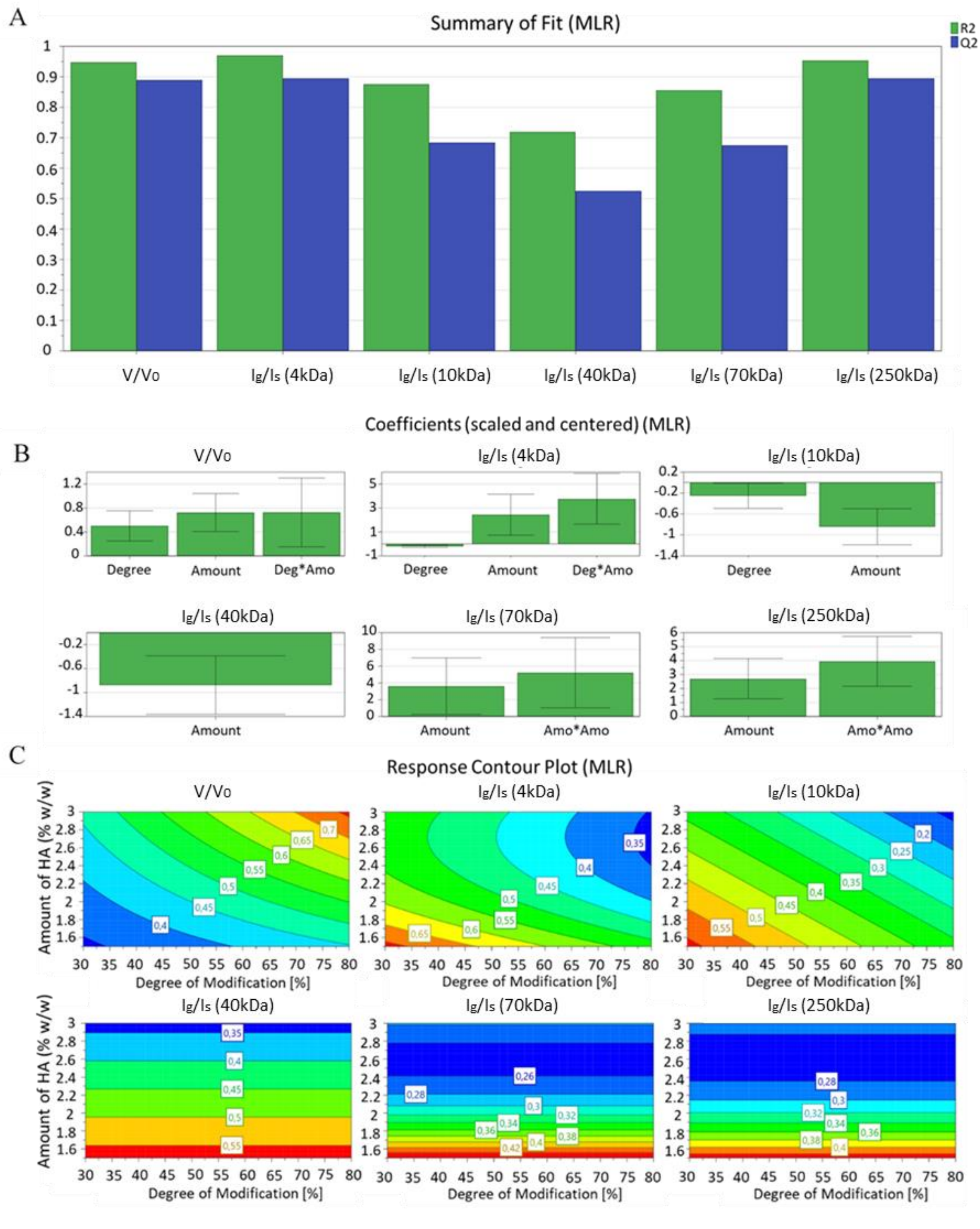


**Figure S11**. Statistical MLR analysis of the experimental results. A: Summary of fit plot for models describing the volume ratio $V/V_0$ and fluorescence intensity ratio $I_g/I_s$ of the microgels. B: Coefficient plots showing the effect of the two factors (degree of ethylacrylamide modification of HA and amount of HA in solution during gel production) on the $V/V_0$ and $I_g/I_s$ of the gels, confidence interval 95%. C: Response contour plot describing the same relationship as in figure B.

## S6. $^{1}$HNMR spectra for ethylacrylamide functionalized HA polymer chains

The degree of ethylacrylamide functionalization/modification of the HA polymer chains used for the microgels was determined by $^1$H NMR at 25 Cº in $D_2O$. The resonance of the protons at 1.9 ppm (corresponding to protons of the N-acetyl group of Hyaluronic acid) was used for normalization. Proton resonances at 5.6 and 6.1 confirmed the presence of the introduced ethylacrylamide groups, respectively. Below are the $^1$H NMR spectra for the 4 different synthesis batches including detailed amounts of materials used for each synthesis, the signal at 4.7 ppm in all spectra corresponds to water.

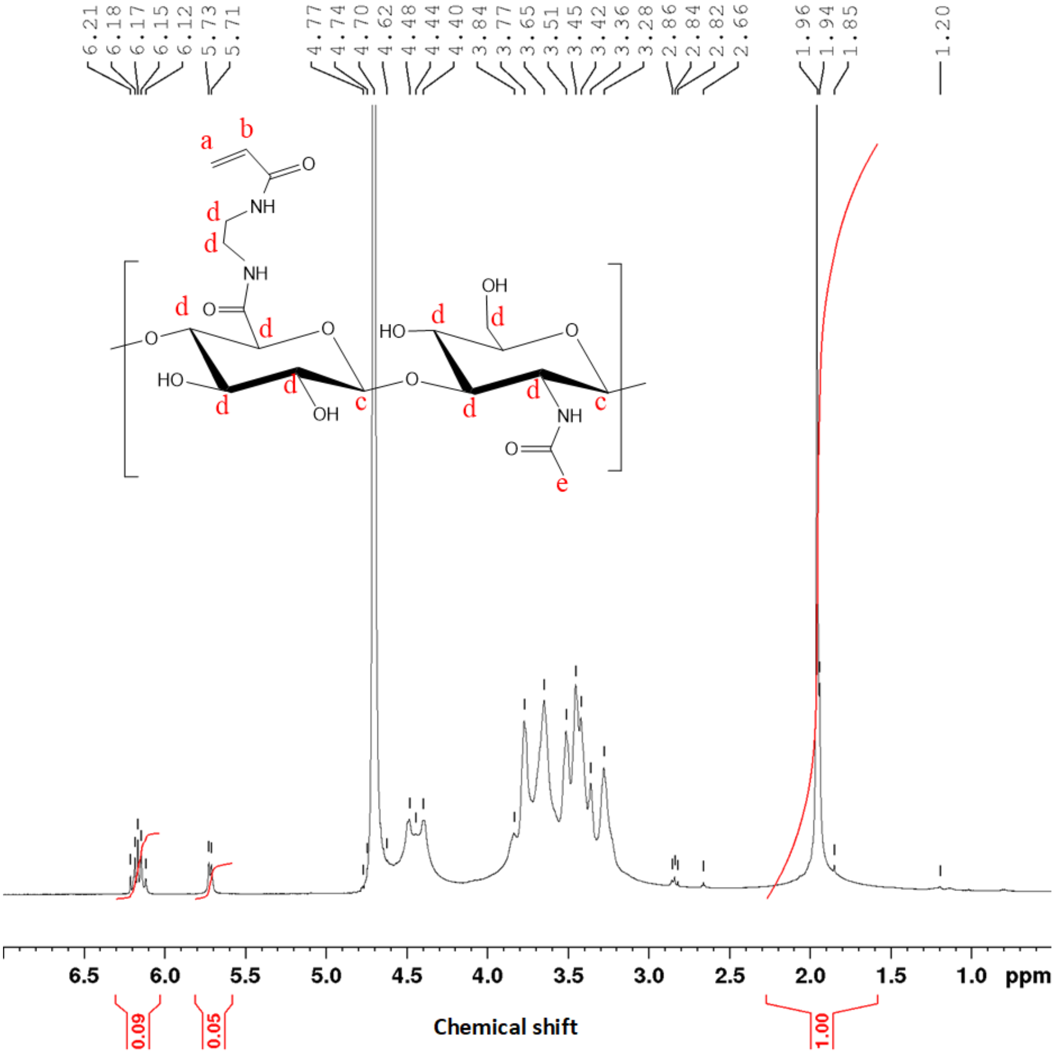


**Figure S12.** $^1$H NMR spectra of ethylacrylamide modification HA batch 1.

**Table S3.** Weight (mg), amount (mol) and equivalent ratios of materials used during the ethylacrylamide modification of HA batch 1, also targeted degree of modification ($f_0$) and degree of modification experimentally determined by $^1H$ NMR (f).

| Material | $M_w$ (g/mol) | Weight (mg) | Amount (mol) | Equivalent ratio | targ. DOF | DOF/NMR |
|---|---|---|---|---|---|---|
| Sodium hyaluronate b5 | 380 | 200 | 0.000526 | 1.0000 | | |
| Acrylamide crosslinker | 150.6 | 43 | 0.000286 | 0.5425 | 50%* | ~13 % |
| HOBt | 153 | 76 | 0.000497 | 0.9438 | | |
| EDC | 191.7 | 143.4 | 0.000748 | 1.4213 | | |

*Reaction time: 8 h instead of 24 h

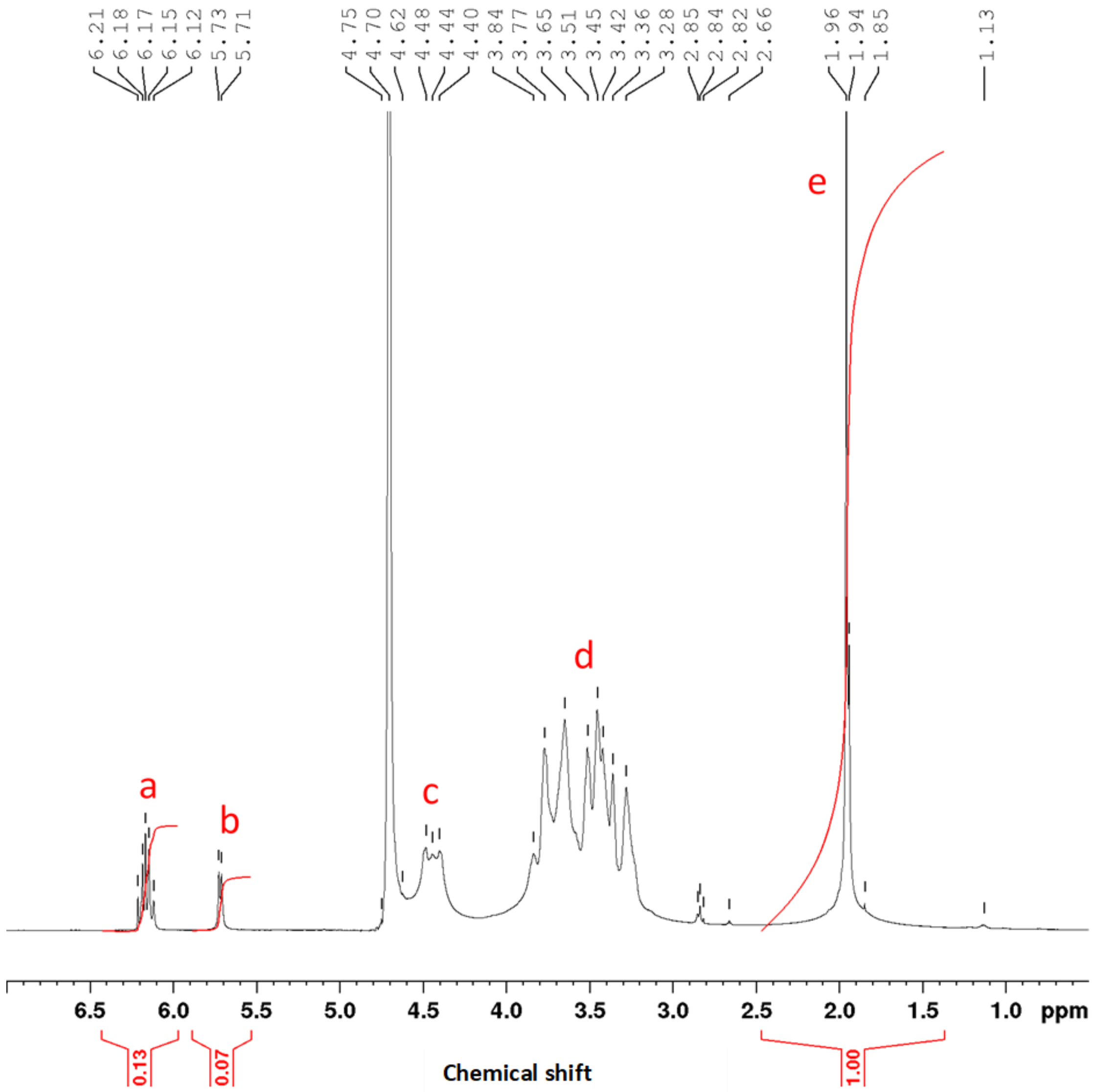


**Figure S13.** $^1H$ NMR spectra of ethylacrylamide modification HA batch 2.

**Table S4**. Weight (mg), amount (mol) and equivalent ratios of materials used during the ethylacrylamide modification of HA batch 2, also targeted degree of modification ($f_0$) and degree of modification experimentally determined by $^{1}$H NMR (f).

| Material | $M_w$ (g/mol) | Weight (mg) | Amount (mol) | Equivalent ratio | targ. DOF | DOF/NMR |
|---|---|---|---|---|---|---|
| Sodium hyaluronate b2 | 380 | 400 | 0.001053 | 1.0000 | | |
| Acrylamide crosslinker | 150.6 | 105 | 0.000697 | 0.6624 | ca 66 % | ~21% |
| HOBt | 153 | 152 | 0.000993 | 0.9438 | | |
| EDC | 191.7 | 287 | 0.001497 | 1.4223 | | |

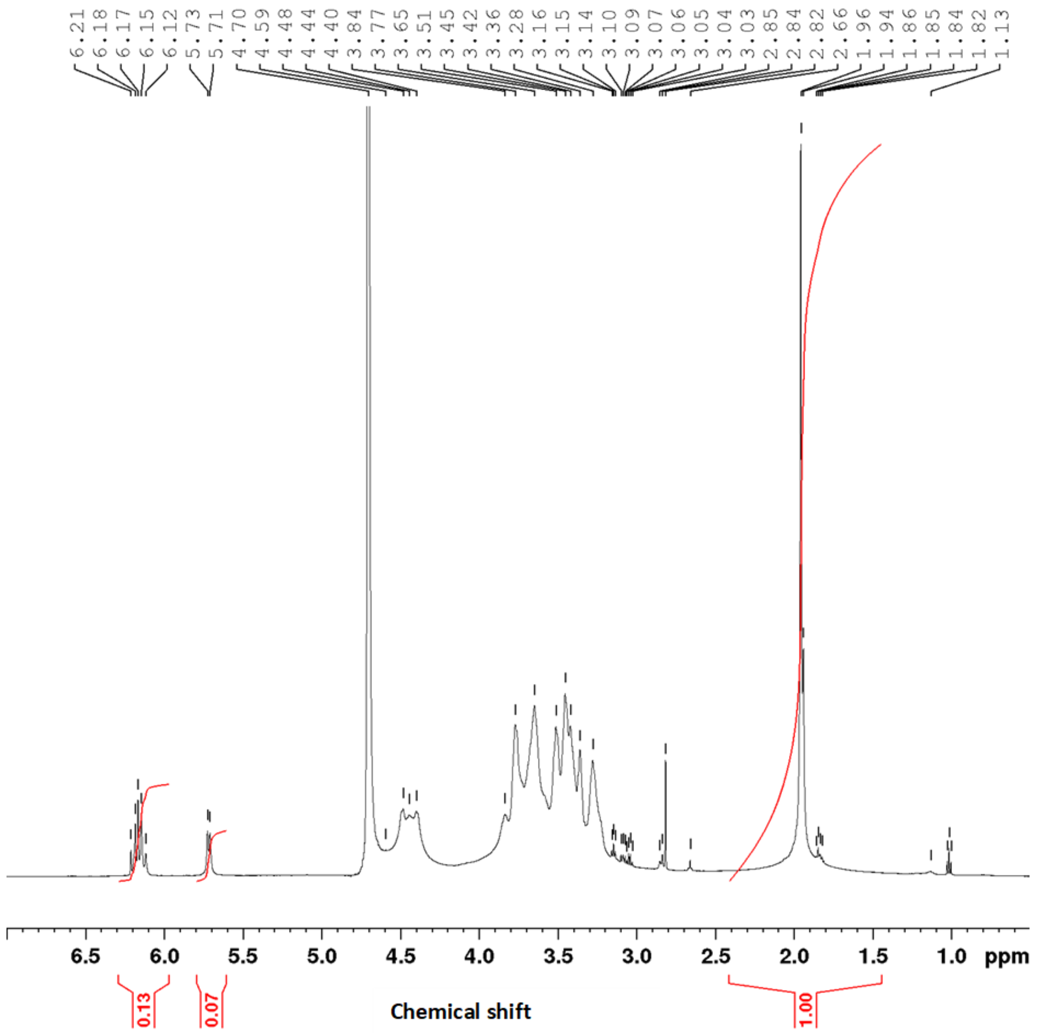


**Figure S14.** $^{1}$H NMR spectra of ethylacrylamide modification HA batch 3.

**Table S5.** Weight (mg), amount (mol) and equivalent ratios of materials used during the ethylacrylamide modification of HA batch 3, also targeted degree of modification ($f_0$) and degree of modification experimentally determined by $^{1}$H NMR ($f$).

| Material | $M_w$ g/mol) | Weight (mg) | Amount (mol) | Equivalent ratio | targ. DOF | DOF/NMR |
|---|---|---|---|---|---|---|
| Sodium hyaluronate b4 | 380.0 | 400.1 | 0.001053 | 1.0000 | | |
| Acrylamide crosslinker | 150.6 | 87.2 | 0.000579 | 0.5499 | ca 55 % | ~21 % |
| HOBt | 153.0 | 151 | 0.000987 | 0.9373 | | |
| EDC | 191.7 | 283 | 0.001476 | 1.4021 | | |

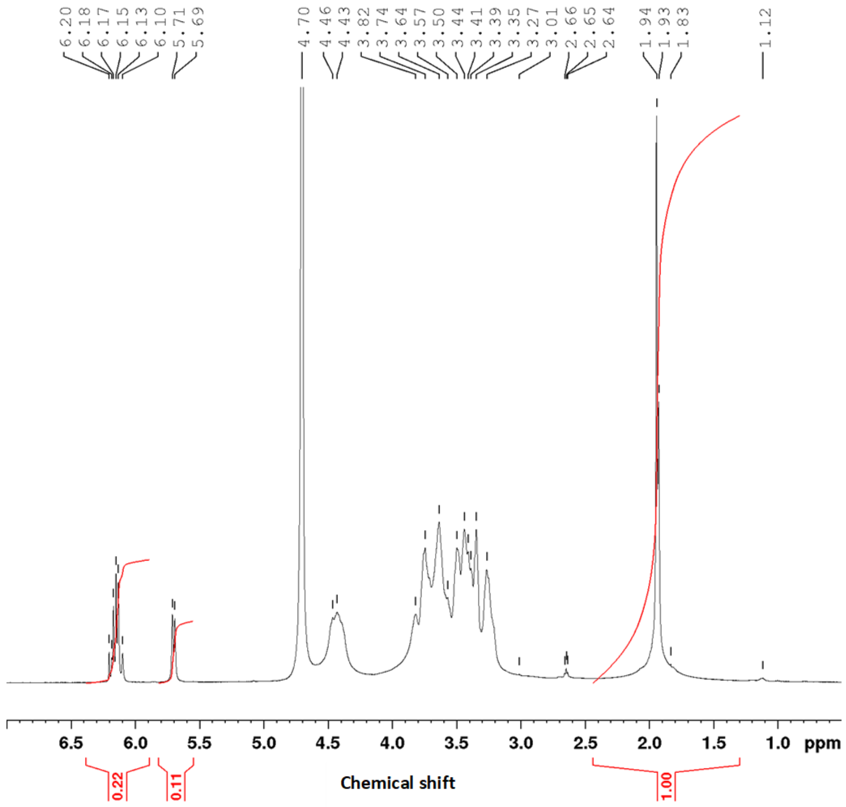


**Figure S15.** $^{1}$H NMR spectra of ethylacrylamide modification HA batch 4.

**Table S6.** Weight (mg), amount (mol) and equivalent ratios of materials used during the ethylacrylamide modification of HA batch 4, also targeted degree of modification ($f_0$) and degree of modification experimentally determined by $^{1}$H NMR ($f$).

| Material | $M_w$ (g/mol) | Weight (mg) | Amount (mol) | Equivalent ratio | targ. DOF | DOF/NMR |
|---|---|---|---|---|---|---|
| Sodium hyaluronate b3 | 380 | 401.4 | 0.001056 | 1.0000 | | |

| | | | | | | | |
|---|---|---|---|---|---|---|---|
| Acrylamide crosslinker | 150.6 | 117 | 0.000777 | 0.7355 | | ca 70 % | ~33 % |
| HOBt | 153 | 154 | 0.001007 | 0.9529 | | | |
| EDC | 191.7 | 287 | 0.001497 | 1.4173 | | | |
